# Ischemic injury of the upper gastrointestinal tract after out-of-hospital cardiac arrest: a prospective, multicenter study

**DOI:** 10.1186/s13054-022-03939-9

**Published:** 2022-03-14

**Authors:** D. Grimaldi, S. Legriel, N. Pichon, P. Colardelle, S. Leblanc, F. Canouï-Poitrine, O. Ben Hadj Salem, G. Muller, N. de Prost, S. Herrmann, S. Marque, A. Baron, B. Sauneuf, J. Messika, M. Dior, J. Creteur, J. P. Bedos, E. Boutin, A. Cariou

**Affiliations:** 1 Department of Intensive Care CUB-Erasme, Route de Lennik, 808, Université Libre de Bruxelles (ULB), 1070 Brussels, Belgium; 2 AfterROSC Network Group, Paris, France; 3 Medico-Surgical Intensive Care Unit, Versailles Hospital, Le Chesnay, Paris, France; 4 Medico-surgical Intensive Care Unit, General Hospital Center, Brive-la-Gaillarde, France; 5 Gastroenterology, C.H. Versailles, Le Chesnay, France; 6 Gastroenterology, APHP, Hôpital Cochin, Paris, France; 7 Unité de Recherche Clinique (URC Mondor), Assistance Publique des Hôpitaux de Paris (AP-HP), Hôpitaux Universitaires Henri Mondor, Créteil, France; 8 INSERM, IMRB, Equipe CEpiA (Clinical Epidemiology and Ageing), University Paris Est Creteil, Créteil, France; 9 Intensive Care Unit, Centre Hospitalier Intercommunal Meulan - Les Mureaux, Meulan en Yvelines, France; 10 ICU, Centre Hospitalier Régional Orleans, Orléans, France; 11 Medical Intensive Care Unit, Hôpitaux Universitaires Henri-Mondor, Assistance Publique – Hôpitaux de Paris (AP-HP), Créteil, France; 12 Université Paris-Est Créteil Val de Marne, Créteil, France; 13 Groupe de Recherche Clinique CARMAS, Université Paris Est-Créteil, Créteil, France; 14 Gastro-enterology, Centre Hospitalier Régional d’Orleans, Orléans, France; 15 ICU, Centre Hospitalier Sud Francilien, Corbeil-Essonnes, France; 16 Gastroenterology, Centre Hospitalier Sud Francilien, Corbeil-Essonnes, France; 17 ICU, Chpc – Centre Hospitalier Public Du Cotentin : Hospital Louis Pasteur, Cherbourg-en-Cotentin, France; 18 APHP.Nord-Université de Paris, Medico-surgical ICU, Hôpital Louis Mourier, Colombes, France; 19 INSERM, PHERE UMRS 1152, Université de Paris, Paris, France; 20 DMU ESPRIT, Department of Gastroenterology, AP-HP, Hopital Louis Mourier, 92700 Colombes, France; 21 Medical Intensive Care Unit, Cochin University Hospital (APHP), Paris, France; University of Paris - Medical School, Paris, France; 22 University of Paris - Medical School, Paris, France

**Keywords:** Cardiac arrest, Gastroscopy, Gastrointestinal tract, Gut, Ischemia/reperfusion, Mesenteric ischemia, Organ failure

## Abstract

**Background:**

The consequences of cardiac arrest (CA) on the gastro-intestinal tract are poorly understood. We measured the incidence of ischemic injury in the upper gastro-intestinal tract after Out-of-hospital CA (OHCA) and determined the risk factors for and consequences of gastrointestinal ischemic injury according to its severity.

**Methods:**

Prospective, non-controlled, multicenter study in nine ICUs in France and Belgium conducted from November 1, 2014 to November 30, 2018. Included patients underwent an esophago-gastro-duodenoscopy 2 to 4 d after OHCA if still intubated and the presence of ischemic lesions of the upper gastro-intestinal tract was determined by a gastroenterologist. Lesions were a priori defined as severe if there was ulceration or necrosis and moderate if there was mucosal edema or erythema. We compared clinical and cardiac arrest characteristics of three groups of patients (no, moderate, and severe lesions) and identified variables associated with gastrointestinal ischemic injury using multivariate regression analysis. We also compared the outcomes (organ failure during ICU stay and neurological status at hospital discharge) of the three groups of patients.

**Results:**

Among the 214 patients included in the analysis, 121 (57%, 95% CI 50–63%) had an upper gastrointestinal ischemic lesion, most frequently on the fundus. Ischemic lesions were severe in 55/121 (45%) patients. In multivariate regression, higher adrenaline dose during cardiopulmonary resuscitation (OR 1.25 per mg (1.08–1.46)) was independently associated with increased odds of severe upper gastrointestinal ischemic lesions; previous proton pump inhibitor use (OR 0.40 (0.14–1.00)) and serum bicarbonate on day 1 (OR 0.89 (0.81–0.97)) were associated with lower odds of ischemic lesions. Patients with severe lesions had a higher SOFA score during the ICU stay and worse neurological outcome at hospital discharge.

**Conclusions:**

More than half of the patients successfully resuscitated from OHCA had upper gastrointestinal tract ischemic injury. Presence of ischemic lesions was independently associated with the amount of adrenaline used during resuscitation. Patients with severe lesions had higher organ failure scores during the ICU stay and a worse prognosis.

*Clinical Trial Registration* NCT02349074.

**Supplementary Information:**

The online version contains supplementary material available at 10.1186/s13054-022-03939-9.

## Introduction

The prognosis of patients successfully resuscitated from out-of-hospital cardiac arrest (OHCA) is influenced by the development of potentially lethal post-cardiac arrest (CA) organ failure [1–3]. Indeed, the whole-body ischemia–reperfusion experienced during CA induces organ injury that can be worsened by post-CA shock as a result of the secretion of pro-inflammatory cytokines and the presence of endotoxemia [4, 5]. Among the different organs, injury to the gastrointestinal (GI) tract has been poorly studied but could be a determinant of subsequent multi-organ failure.

Evidence for injury of the GI tract after CA is suggested by the high frequency of abnormalities in biomarkers of GI function, such as citrulline or intestinal fatty-acid binding protein, and by the frequency of endotoxemia observed in these patients [6, 7]. The most severe form of intestinal ischemia results in transmural necrosis, a pathology named non-occlusive mesenteric ischemia (NOMI). NOMI has been reported in less than 5% of patients successfully resuscitated after CA [8]. However, this entity captures only transmural necrosis and can be difficult to diagnose. Thus the real incidence of ischemic GI lesions, their risk factors and their potential involvement in post-CA shock is unknown.

We therefore conducted a prospective, multicenter study to determine the incidence of upper GI ischemic lesions after OHCA, and to analyze risk factors and association with post-CA organ failure. Our primary objective was to determine the incidence of macroscopic lesions of the upper GI tract after successfully resuscitated OHCA by performing a gastroscopy during the ICU stay. Our main secondary aims were 1) to describe the location and the severity of such lesions; 2) to determine which patient and CA characteristics were associated with presence of upper GI lesions; and 3) to determine the association between the severity of the GI lesions and the severity of post-CA organ failure and survival without major neurological sequelae.

## Methods and design

ENTRACT was a prospective, multicenter, observational cohort study in patients admitted to nine ICUs in France and Belgium between November 1, 2014 to November 30, 2018. Approval was obtained from an independent ethics committee in both countries (Comité de Protection des Personnes CPP Ile de France XI, #14059 and Ethic Committee Erasme Hospital P2016/319). The study was registered at clinicaltrials.gov NCT02349074.

The trial complied with the Declaration of Helsinki and Good Clinical Practices, and French regulatory requirements. The patients were unable to provide informed consent at inclusion so written informed consent was obtained from their surrogates before inclusion; a written informed consent was obtained from the patients when they regained consciousness, in compliance with French and Belgian laws.

### Patients

All adult patients (> 18 years) admitted after OHCA to one of the participating ICUs after OHCA were screened for eligibility by the ICU physicians. Patients who were hospitalized within the first 5 days after OHCA, had a temperature > 36 °C at the time of enrollment, and still required endotracheal intubation and mechanical ventilation were considered for inclusion. Exclusion criteria were in-hospital CA, extubation before gastroscopy, any suspicion of GI tract perforation, severe bleeding diathesis despite transfusion of coagulation products, patients with cardiac valver prostheses or previous endocarditis, suspicion of Creutzfeldt-Jakob disease, pregnancy or breast-feeding, and absence of medical insurance or being under guardianship (according to French legislation). If a patient had a severe coagulation disorder (platelet count < 30 G/L, International Normalized Ratio > 2) or was receiving heparin treatment or combined platelet inhibition treatment, but did not have intractable bleeding, inclusion was possible but GI biopsies were not performed.

### Examination

Patients included in the study systematically underwent esophago-gastro-duodenoscopy (hereafter called gastroscopy) during their ICU stay after rewarming and before day-5. The clinicians were aware of the results of the gastroscopy and were free to modify the treatment accordingly.

Targeted temperature management to between 33 and 36 °C was strongly advised. Hemodynamic management was protocol driven. Briefly, if the mean blood pressure decreased under 65 mmHg, or if there were clinical signs of reduced tissue perfusion, fluid responsiveness was assessed (choice of method was left to the individual center); in case of hypovolemia, a crystalloid solution was infused until resolution. If the patient was fluid unresponsive, transthoracic echocardiography and/or invasive cardiac output monitoring were performed to assess need for inotropic agents. Vasopressor dose was titrated to maintain MAP between 65 and 75 mmHg. Other treatments were left to the treating physician’s discretion.

### Data collection and definitions

Data regarding CA were collected according to Utstein style [9]. History of previous cardiovascular or GI disease and previous use of Proton Pump Inhibitors (PPI) or Non-steroidal anti-inflammatory drugs were noted.

Hypothermia was defined as a body temperature < 34 °C for at least 12 h within the first 24 h.

GI symptoms before gastroscopy were defined a priori as vomiting, GI bleeding, bloody or mucoid diarrhea and feeding intolerance. PPI administration and enteral feeding were collected.

Post-CA shock was defined as the need for continuous vasopressor infusion for more than 6 h despite adequate fluid resuscitation in the first 48 h after ICU admission [10]. The duration of shock was evaluated using the number of vasopressor-free days at day 10 [11] i.e. the number of days alive without vasopressors.

### Outcomes

The primary outcome measure was the presence of macroscopic ischemic lesions in the upper GI tract, and was determined during gastroscopy by the gastroenterologist from each center.

Secondary outcomes were the location (esophagus, fundus, antrum, duodenum) and the severity of the lesions; the presence of post-CA shock, the SOFA score [12] during the first 3 days and at day-5 and 8 following CA; the ICU and hospital mortality; and the neurological outcome at hospital discharge assessed using the Cerebral Performance Category (CPC) scale.

The gastroenterologist was asked to complete a standardized form describing the results of the gastroscopy in the four locations. For mucosal lesions, the gastroenterologist was asked to state whether the lesions were ischemic or due to another cause. An ischemic mechanism was suspected if the mucosa was pale and inflamed, there was diffuse or giant ulceration not of a peptic origin, or there was necrosis. The severity of ischemic lesions was a priori classified as moderate if there was erythema or edema, and severe if there was ulceration, localized necrosis, or extensive necrosis [13]. Patients were classified according to the most severe lesion that was observed regardless of the location and the presence of other less severe lesions.

The neurological outcome was graded using the Cerebral Performance Category scale at hospital discharge (ranging from 1 [no or minor sequelae] to 5 [brain death]); a score of 1 or 2 was considered as a good neurological outcome [14].

### Statistical analysis

Based on the only published study that has reported gastroscopy data following CA [15], we hypothesized that the incidence of upper GI lesions would be 20%. The estimated number of patients to include with a precision of 5% was therefore 246. Based on the hypothesis that gastroscopy would not be performed in 10% of the cases, we therefore planned to enroll 270 patients.

The incidence of ischemic injury at gastroscopy was calculated (number of new cases/ number of exposed patients) by using binomial proportions and the 95% confidence interval (95%CI) was estimated from the normal approximation of a binomial assumption. Patients characteristics and outcomes are given as mean (SD) or median (25th–75th centiles) values, or numbers (%).The three groups (moderate, severe or no ischemic lesions) were compared using an ANOVA or Kruskal–Wallis test, as appropriate, for quantitative variables, and the χ^2^ test for qualitative variables.

To assess risk factors for GI ischemic injury, associations were tested using multinomial logistic regression and estimated using crude and adjusted odds ratios (cORs and aORs, respectively) and their 95% confidence intervals. Candidate variables for the multivariate analysis were those associated with a *p* value < 0.20 in univariate analysis. Pairwise analyses were done to assess confounding factors and candidate variables were removed step-by-step to form the most parsimonious multivariate model with variables associated with ischemic injury with a *p* value < 0.05 (significant) or trend (p value between 0.05 and 0.10). We explored a potential center effect by adding the center in multilevel logistic regression models.

Survival with good neurologic outcome defined (CPC 1 or 2) at hospital discharge was estimated using the Kaplan–Meier method, and survival curves were compared according to the presence or absence of ischemic injury using the log-rank test. Factors associated with outcome were identified using univariate Cox proportional hazard models. The results are expressed as hazard ratios (HRs) and their 95% confidence intervals. We used multivariate modeling to determine whether ischemic injury was associated with outcome, taking into account the variables associated with a *p* value < 0.20 in univariate analysis. Pairwise analyses were done to assess confounding factors, and interactions were sought. The proportional hazards assumption was assessed statistically using the Schoenfeld residuals test. We verified the specification of the model with the Pregibon test (linktest command) to verify that any additional independent variables should be significant by chance. We bootstrap the multivariable model as a sensitivity analysis.

Analyses of the change in SOFA score over time were based on mixed linear regression models with random intercepts for repeated measurements. Factors associated with SOFA score changes were identified using univariate mixed linear regression models. The results are expressed as regression coefficients (β) with 95% confidence intervals. We explored a potential center effect by adding a third level to the mixed linear regression.

All tests were two-tailed and *p* values < 0.05 were considered statistically significant. Missing data were not imputed. Analyses were performed with STATA v15.0 (StataCorp, College Station, TX, USA).

## Results

### Study patients

**Fig. 1:**
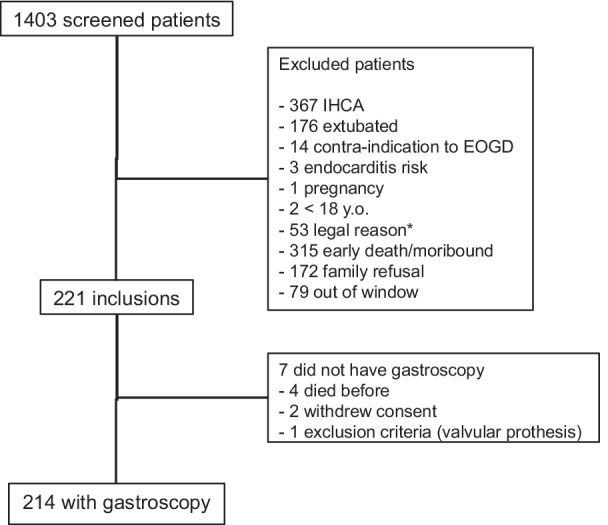
Flow chart of the study. IHCA: intra-hospital cardiac arrest, OGD: Oso-gastro-duodenoscopy. *According to French law: no social insurance, under guardianship, no known identity

Between November 1, 2014 and November 30, 2018, 221 patients were included (Fig. 1). At this point, the sponsor decided to end the study for lack of funding. Among the 221 patients, 214 underwent a gastroscopy.

Patient and CA characteristics are shown in Table 1. The patients were mostly men, with a mean age of 62 (± 14) years. The CA was witnessed in 192/214 (90%) of cases; the initial rhythm was ventricular tachycardia or ventricular fibrillation in 112/214 (52%) patients. The median time from collapse to cardiopulmonary resuscitation (CPR) was 5 [IQR, 0–9] min and from CPR to return of spontaneous circulation (ROSC) was 21 [IQR, 15–30] min. A total of 207/214 (97%) patients were comatose (GCS < 9) at ICU admission. The median lactate concentration was 4.1 [IQR, 2.2–7.4] mEq/L.

**Table 1:** Patient characteristics before gastroscopy according to the presence of upper gastrointestinal tract ischemic injury

	No lesions *N* = 93	Moderate lesions *N* = 66	Severe lesions *N* = 55	p*
Age (years), med [IQR]	66 [58–72]	58 [47–71]	64 [55–75]	0.05
Women, N (%)	29 (31)	15 (23)	12 (22)	0.34
Peripheral artery disease, N (%)	13 (14)	4 (6)	2 (4)	0.08
Coronaropathy, N (%)	16 (17)	9 (14)	7 (13)	0.71
PPI before CA, N (%)	29 (31)	11 (17)	8 (15)	0.03
Witnessed CA, N (%)	87 (94)	59 (89)	46 (84)	0.16
No flow (min), med [IQR missing: * N* = 34	4 [0–10]	5 [2–5]	5 [2–10]	0.21
Low flow (min), med [IQR], missing: * N* = 3	19 [12–30]	22 [15–30]	22 [16–35]	0.10
VF/VT, N (%), missing: * N* = 4	47 (50)	40 (61)	25 (45)	0.2
Adrenaline (mg), med [IQR]	1 [0–3]	2 [1–4]	3 [1–5]	0.01
Shock (number), med [IQR], missing: * N* = 4	2 [0–4]	3 [1–4]	2 [0–5]	0.31
Lactate level at ICU admission (meq/L), med [IQR]	3.9 [2.1–6.8]	3.7 [2.4–6.5]	4.9 [2.2–9.5]	0.28
SAPS-2, mean (SD), missing: * N* = 27	85.0 (± 9.1)	83.3 (± 9.4)	86.4 (± 10.6)	0.28
SOFA score^†^, med [IQR], missing: * N* = 7	11 [9–13]	11 [9–13]	11 [9–13]	0.43
Hematocrit^†^ (%), med [IQR]	39 [35–44]	43 [37–47]	42 [37–48]	0.01
Serum bicarbonate^†^ (meq/L), med (IQR)	18.1 [15.5–21.3]	19.4 [17.1–22.2]	18.1 [14–22.2]	0,09
Serum chloride^$^ (meq/L), med [IQR]	107 [104–110]	106 [104–109]	104 [102–108]	0.07
T° < 34 °C^†^, N (%)	46 (49)	27 (41	27 (49)	0.52
PPI during ICU stay, N (%)	73 (78)	54 (82)	42 (76)	0.77
Enteral nutrition, N(%) missing:* N* = 18	38 (47)	25 (39)	18 (35)	0.31
Gastrointestinal symptoms, N (%): missing: * N* = 1	16 (17)	8 (12)	14 (26)	0.16

PPI: proton pump inhibitors, VF: ventricular fibrillation, VT: ventricular tachycardia

^†^First 24 h of ICU stay

^$^Between 24 and 48 h of ICU stay

*P value was obtained by comparison of variables across the 3 groups using chi-2 or Fisher exact test for qualitative variables and ANOVA or Kruskal–Wallis test for quantitative variables

### Gastroscopy findings (Fig. 2, Tables 1 and 2)

**Fig. 2:**
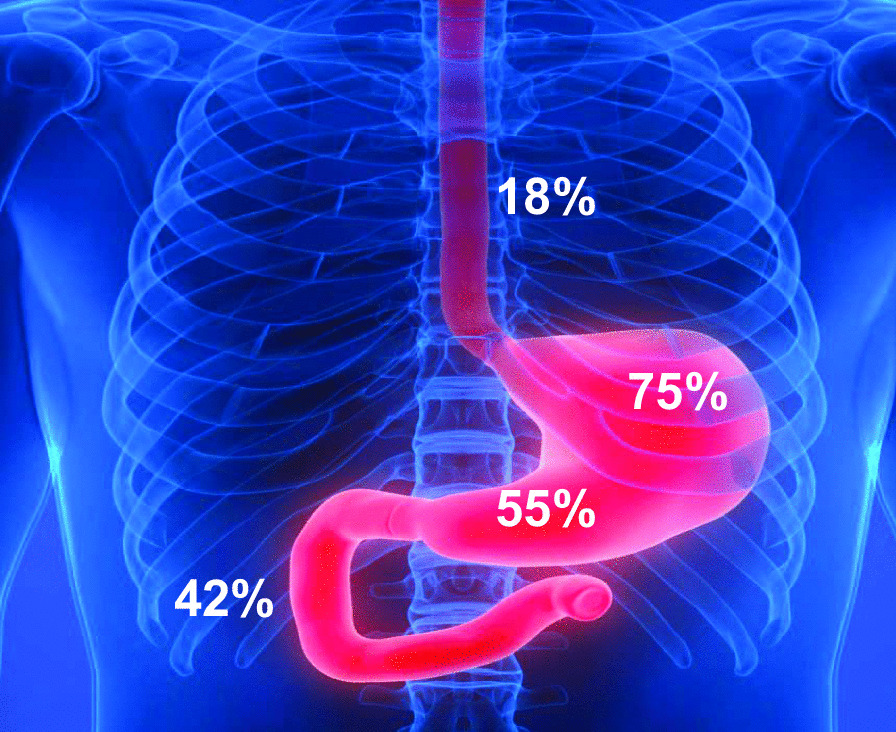
Repartition of the ischemic lesions across the upper gastrointestinal tract

Among the 214 patients analyzed, 121 had at least one ischemic upper GI lesion; the incidence of ischemic injury was 56.5% (CI95%: 50.2%-63.1%). The location of the ischemic lesions, primarily in the fundus, is shown in Fig. 2. The lesions were moderate (edema, erythema) in 67/121 patients (55%) and severe in 54/121 (45%) including 10 patients with localized necrosis and 44 with ulceration (Additional file 1). There were no cases of extensive necrosis. The proportion of ischemic lesions was similar regardless of the timing of the gastroscopy post-CA (data not shown). Additional file 2 indicates the treatment advised by the gastroenterologist performing the gastroscopy, double dose of PPI was more frequently advised in patients with ischemic lesions.

### Patient characteristics according to the presence and severity of upper GI tract ischemic injury (Tables 1 and 2)

Patient characteristics according to the severity of ischemic lesions are shown in Table 1. Patients with ischemic lesions were less likely to be taking proton pump inhibitors (PPI) before CA than those without lesions. Regarding the CA characteristics, no-flow and low-flow times did not differ significantly between the groups. Patients with ischemic lesions received significantly higher doses of adrenaline during cardiopulmonary resuscitation (CPR) than those without ischemic lesions. Disease severity at ICU admission, as assessed by lactate level, SAPS-2 and SOFA scores, was similar across groups. On day 1, the hematocrit was higher in patients with ischemic lesions than in those without; this was the only biological variable that differed between the groups. Of note, neither ICU administration of PPI, nor enteral feeding was associated with GI ischemic injury. In the multivariate logistic regression analysis, higher adrenaline dose used during CPR was the only variable associated with a greater odd of moderate and severe ischemic lesions; previous PPI use and day 1 serum bicarbonate were associated with a reduced odds of severe GI ischemic injury (Table 2). When we repeated the multivariate analysis, by using multilevel logistic regression, with the center at the level 2 there was no significant center effect when comparing this model to the logistic regression model (*p* = 0.40 for severe lesions and *p* = 0.46 for moderate lesions), the rough number for each centers are shown in the Additional file 3. The multivariable model was correctly specified (*p* = 0.87). In a bootstrap sensitivity analysis, the results were similar except that the association between peripheral arterial disease and ischemic lesions became non-significant (*p* = 0.77).

**Table 2:** Multivariate analysis of factors associated with upper gastrointestinal tract ischemic lesions

	aOR moderate lesions* [IC95%]	*p*	aOR severe lesions* [IC95%]	*p*
Peripheral arterial disease	0.39 [0.11–1.41]	0.15	0.20 [0.04–1.05]	0.06
Previous PPI	0.50 [0.21–1.18]	0.11	0.40 [0.14–1.00]	0.05
Adrenaline dose/mg	1.21 [1.04–1.40]	0.01	1.25 [1.08–1.46]	0.004
Hematocrit † /%	1.12 [1.03–1.22]	0.009	1.06 [0.97–1.16]	0.2
Bicarbonate† /meq/L	0.95 [0.88–1.03]	0.24	0.89 [0.81–0.97]	0.007
Chloremia^$^ /meq/L	1.05 [1.00–1.10]	0.07	1.05 [1.00–1.10]	0.06

*n* = 211 (3 missing data)

*Adjusted Odd Ratio (aOR) obtained from multivariate logistic regression, standard group = absence of ischemic lesions. PPI: proton pump inhibitors

^†^First 24 h of ICU stay

^$^Between 24 and 48 h of ICU stay

### Patient outcomes (Fig. 3, Table 3, Additional files 2 and 3)

**Fig. 3:**
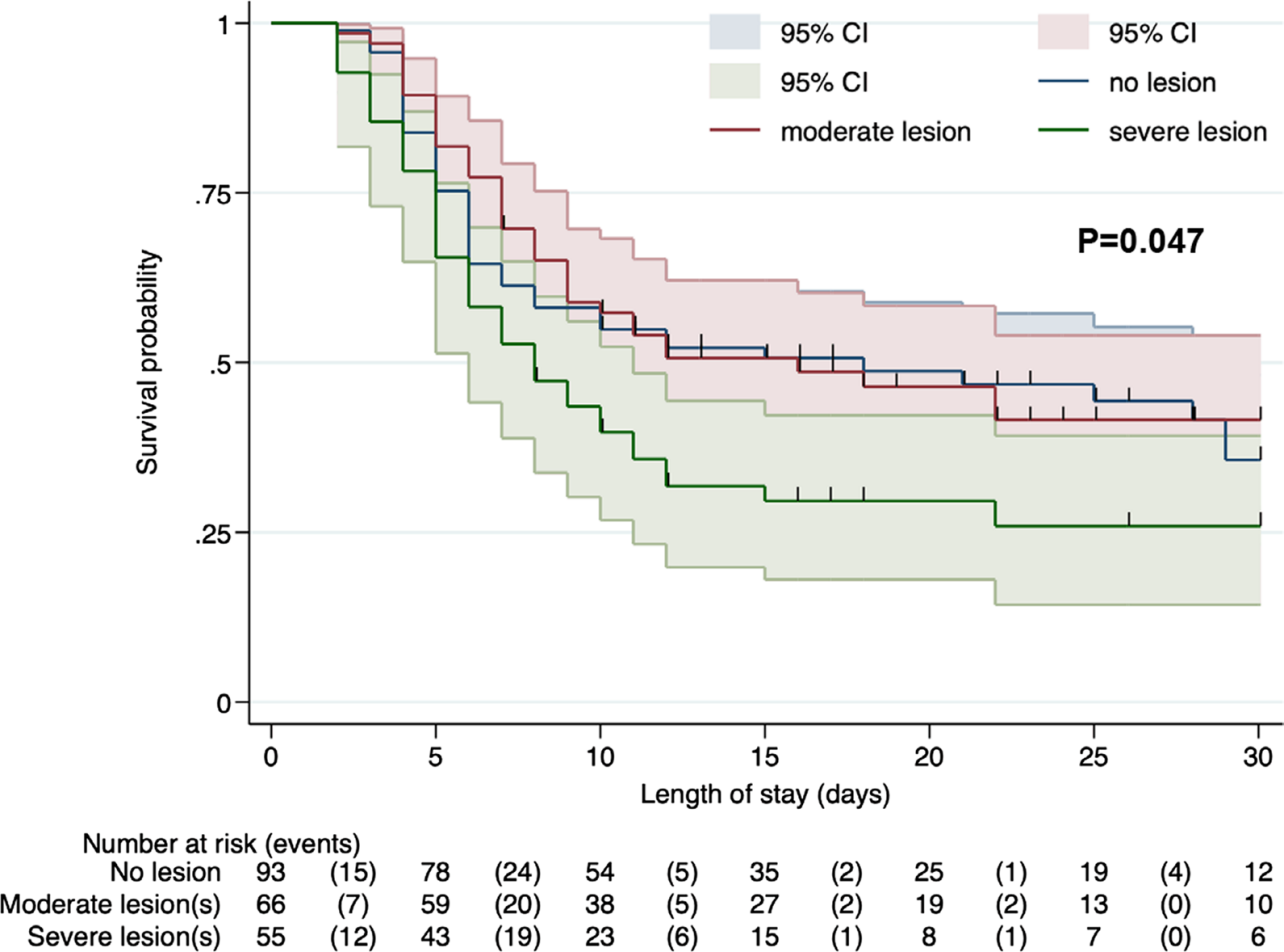
Survival without neurological sequelae (defined as a CPC score of 1 or 2) according to the presence and severity of ischemic lesions. Comparison performed using the log rank test

**Table 3:** Patient outcomes according to the presence of ischemic upper gastrointestinal tract lesions

	No lesions *N* = 93	Moderate lesions *N* = 66	Severe lesions *N* = 55	p*
Post-resuscitation shock, N (%)	61 (65.6)	39 (59)	38 (69)	0.50
Day 10 Vasopressors free days, med (IQR), missing: * N* = 16	5 [2–7]	5 [2–8]	4 [2–7]	0.78
Gastrointestinal hemorrhage, N (%), missing: * N* = 1	5 (5.4)	0 (0)	5 (9.1)	0.03
ICU-mortality, N (%), missing: * N* = 1	46 (50)	33 (50)	39 (71)	0.02
Hospital mortality, N (%)	50 (54)	35 (53)	39 (71)	0.08
CPC 1 or 2 at hospital discharge, N (%), missing: * N* = 1	38 (41)	29 (44)	15 (27)	0.14

CPC: Cerebral Performance Category

*P value was obtained by comparison of variables using chi-2 or Fisher exact test for qualitative variables and Kruskal–Wallis test for quantitative variables, as appropriate

Patients with severe ischemic GI lesions had a similar SOFA score on ICU admission to that of patients without lesions, but a higher SOFA scores on days 3 and 8 post-CA (Additional files 4 and 5). There was no center effect in the multilevel model. Patients with severe ischemic lesions had higher ICU mortality (Table 3) and were less likely to survive without neurological sequelae (Fig. 3) than patients with no or moderate ischemic lesions.

## Discussion

In this prospective multicenter study, we systematically performed a gastroscopy in patients admitted to the ICU after OHCA. In this cohort, more than 50% of patients had upper GI tract ischemic injury and almost one quarter had severe lesions, defined as ulceration or necrosis. In multivariate analysis, we observed that a higher dose of adrenaline given during CPR was independently associated with the occurrence of upper GI ischemic injury. Patients with severe lesions had higher organ failure scores during the ICU stay, higher ICU mortality rates and were less likely to have a favorable neurological outcome at hospital discharge than patients with no or moderate ischemic lesions.

To our knowledge, our study is the first to prospectively report the incidence of upper GI tract ischemic injury after CA as assessed by systematic gastroscopy (i.e., gastroscopy not performed just for clinical symptoms). In a retrospective single center study that evaluated the results of gastroscopy after OHCA, all 36 included patients had gastric mucosal lesions [15]. The multicenter and prospective nature of our study, as well as the size of the cohort, makes our findings robust with a lower 95% confidence interval of 50%. However, we included only patients admitted to the ICU after OHCA who were still receiving invasive mechanical ventilation 2 to 4 days after resuscitation. Thus, the global incidence of upper GI ischemic injury after OHCA, including patients who die early and patients who are extubated before this time point may, be different. It is likely that these two groups of patients would have opposite effects on the incidence of GI ischemic injury and we do not know whether including them would have markedly changed our findings.

The high incidence of upper GI lesions highlights the gut’s susceptibility to ischemia reperfusion injury as suggested by intestinal biomarkers studies [6, 7], that have indicated that nearly all patients have a certain degree of gut dysfunction after OHCA. Our study thus adds to the previous literature bridging the gap between biomarker studies and the advent of transmural necrosis (NOMI), which has been observed in 1 to 5% of patients [8, 16].

Surprisingly, no-flow or low-flow times were not associated with the occurrence of GI ischemic injury. This may be due to an underrepresentation of patients with more extreme resuscitation times (very short or very long), because these patients are more likely to die in the first 48 h or to no longer be receiving mechanical ventilation. Nevertheless the median low-flow time was still around 20 min with an upper quartile of 30 min, which corresponds to a significant ischemia–reperfusion insult. We observed a lack of association between time from collapse to ROSC and the presence of endotoxemia in an earlier study in patients with OHCA [11]. Our hypothesis to explain this finding is that ischemia–reperfusion is a necessary but not always sufficient condition for GI ischemic injury, but that the susceptibility of the gut is driven by other factors in addition to the duration of the CA. Indeed, in line with our previous work, adrenaline dose was an independent risk factor for the presence of upper GI tract ischemic injury [11]. The differences in median adrenaline dose among the groups was small; however, in the post-cardiac arrest setting, even small differences in adrenaline dose have been observed to be associated with outcome [11, 17, 18]. This effect could be explained by the well-known detrimental effect of adrenaline on mesenteric blood flow [19], and may be one of the reasons underlying the paradoxical negative effects of adrenaline bolus during CPR [20, 21]. The fact that the total dose of adrenaline during CPR differed across groups despite similar low-flow times may be explained by differences in delay between boluses of adrenaline (3 to 5 min in guidelines [22]) and by the duration of bystander CPR. We could not analyze further this finding further as we did not collect these data. Conversely, the finding that previous use of PPI appeared protective, suggests that a low gastric pH at the time of CA may favor development of ischemic lesions. We identified biological factors consistent with metabolic acidosis (low serum bicarbonate, high serum chloride) that may be associated with ischemic lesions although the results from the multivariate analysis were not totally consistent. If this association is confirmed, two hypotheses could be raised: first metabolic acidosis could be an indicator of impaired organ perfusion including the GI tract; second, as the GI ischemic lesions were present before the gastroscopy, the association may indicate that these biological findings were the consequence of the ischemic process and not a cause.

Severe upper GI ischemic injury was associated with worse outcomes. Patients with severe ischemic lesions, although having a similar SOFA score at ICU admission to patients with no or moderate lesions, had a higher SOFA score later during the ICU stay. However, the rate of post-CA shock was similar in patients with and without GI ischemia. Despite a similar duration from collapse to ROSC, the likelihood of achieving a good neurological recovery appeared lower in patients with a severe upper GI ischemic injury in our time-dependent analysis. These associations could reflect a worse ischemic insult in multiple organ systems not captured by no-flow and low-flow times. They may also indicate that GI ischemic injury acts as a motor of organ failure as suggested elsewhere [23] for example through endotoxin translocation [7, 11]. Our study was not designed to decipher these two, non-mutually exclusive explanations.

Interestingly, there were no notable difference in prognosis in patients with moderate lesions (erythema, pallor) compared to those with no ischemic lesion. Our findings, combined with the limited previous literature, support the idea that the association between GI ischemic injury and clinically relevant outcomes depends on the severity of the ischemic lesions: minimal when limited to mucosal ischemia, but associated with increased organ failure and risk of death of 10–15% when mucosal necrosis develops, and associated with a mortality up to 90% for transmural necrosis [8].

Our study was not designed to evaluate an impact on clinical management. Even if the gastroenterologist advised more frequently to double the dose of PPI in patients with ischemic lesions, we did not collect the patients’ actual treatment dose or the actual treatment after gastroscopy. Whether systematic gastroscopy could improve patient management needs further study. Addition of PPI in the ICU and/or early enteral feeding were not associated with the GI ischemic injury, further studies are needed to determine their potential interest in post-resuscitation care.

Our study has some strengths and limitations. The systematic gastroscopies ensured an accurate measurement of incidence in the population of interest independent of GI symptoms, which did not appear to be related to gastroscopy results. Our selection criteria however precluded the inclusion of the most severely ill patients (who had already died) and the least severely ill (extubated by day 3). This selection appears unavoidable in a research program but we can hypothesize that the incidence would not have been dramatically modified by the inclusion of these patients as mentioned earlier. A second limitation is the premature end of enrollment for lack of funding; however, considering the observational nature of the study, the lower than estimated sample size induces only an increase in the size of the confidence interval, which has limited clinical significance. Third, some factors that may be associated with peptic lesions were not collected, such as chronic steroid therapy or PPI doses. Fourth due to limited sample size, multivariate analysis should be seen as exploratory. Finally, we only evaluated the upper GI tract, thus this study does not give a picture of injury across the whole GI tract.

## Conclusion

More than 50% of patients who were successfully resuscitated after OHCA had ischemic injury of the upper GI ischemic tract. Half of these patients had severe lesions (ulcer, necrosis), which were associated with worse organ failure and a decreased probability of survival without neurological sequelae. Further studies are needed to better understand the link between GI ischemic injury and outcome after CA.

## Supplementary Information

**Additional file 1**. Examples of ischemic lesions.

**Additional file 2**. Therapeutic advices after gastroscopy.

**Additional file 3**. Presence and severity of ischemic lesions according to inclusion centres.

**Additional file 4**. Changes in SOFA score at specific time points during the ICU stay in the different group.

**Additional file 5**. Evolution of SOFA score according to severity of gastrointestinal ischemic lesions.

## Data Availability

The datasets used and/or analyzed during the current study are available from the corresponding author on reasonable request.

## Abbreviations

CPC: Cerebral performance categories
CPR: Cardiopulmonary resuscitation
GI: Gastrointestinal
HR: Hazard ratio
ICU: Intensive care unit
INR: International normalized ratio
NOMI: Non-occlusive mesenteric ischemia
NSAIDs: Non-steroidal anti-inflammatory drugs
OHCA: Out-of-hospital cardiac arrest
OR: Odds ratio
PPI: Proton pump inhibitor
ROSC: Return of spontaneous circulation
SD: Standard deviation
SOFA: Sequential organ failure assessment
