# Supplementary material for: Ischemic injury of the upper gastrointestinal tract after out-of-hospital cardiac arrest: a prospective, multicenter study
Source: Crit Care. 2022 Mar 14;26:59. doi: 10.1186/s13054-022-03939-9 (PMC8919548; doi:10.1186/s13054-022-03939-9)

**Additional File 1**

**of the study by Grimaldi et al.**

**Ischemic injury of the upper digestive tract after out-of-hospital cardiac arrest: a prospective, multicentre study**

Example of duodenal ulceration (A) and fundus necrosis (B)


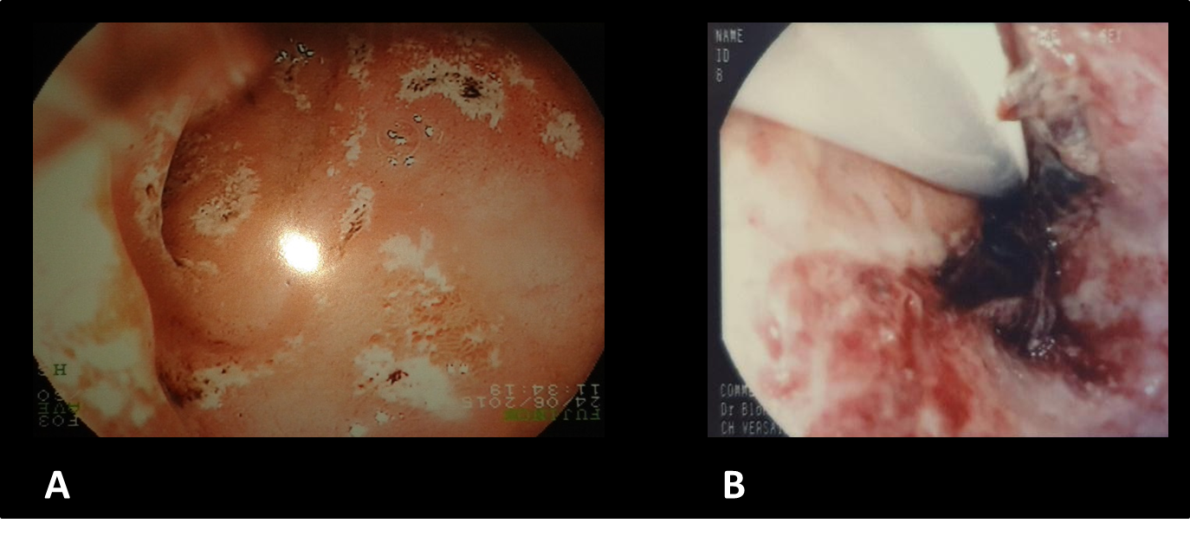

Supplement: Supplementary file 1 — Additional file 1. Examples of ischemic lesions. [file 13054_2022_3939_MOESM1_ESM.docx]
