# Supplementary material for: Ischemic injury of the upper gastrointestinal tract after out-of-hospital cardiac arrest: a prospective, multicenter study
Source: Crit Care. 2022 Mar 14;26:59. doi: 10.1186/s13054-022-03939-9 (PMC8919548; doi:10.1186/s13054-022-03939-9)
Supplement: Supplementary file 2 — Additional file 2. Therapeutic advices after gastroscopy. [file 13054_2022_3939_MOESM2_ESM.docx]

**Additionale File 2**

**of the study by Grimaldi et al.**

**Ischemic injury of the upper digestive tract after out-of-hospital cardiac arrest:**

**a prospective, multicentre study**

Therapeutic advices by the gastroenterologist after the gastroscopy according to the severity of ischemic lesions

|  | No ischemic lesions  N=93 | Moderate lesions  N=66 | Severe lesions  N= 55 | p |
| --- | --- | --- | --- | --- |
| **Starving** | 1 (1) | 9 (14) | 5 (9) | 0.04 |
| **PPI standard dose** | 33 (35) | 40 (61) | 31 (56) | 0.44 |
| **PPI double dose** | 5 (5) | 10 (15) | 19 (35) | 0.004 |
| **Surgeon contact** | 0 (0) | 0 (0) | 0 (0) | na |

The advices were communicated to the physicians in charge of the patients but he remained free to follow them or not. These advices did not take into account the actual treatment of patients

Note that in the “no ischemic lesions” group, patients may have other digestive diseases
