# Supplementary material for: Ischemic injury of the upper gastrointestinal tract after out-of-hospital cardiac arrest: a prospective, multicenter study
Source: Crit Care. 2022 Mar 14;26:59. doi: 10.1186/s13054-022-03939-9 (PMC8919548; doi:10.1186/s13054-022-03939-9)
Supplement: Supplementary file 3 — Additional file 3. Presence and severity of ischemic lesions according to inclusion centres. [file 13054_2022_3939_MOESM3_ESM.docx]

**Additional File 3**

**of the study by Grimaldi et al.**

**Ischemic injury of the upper digestive tract after out-of-hospital cardiac arrest: a prospective, multicentre study**

Presence and severity of ischemic lesions according to inclusion centres

|  | No lesions | Moderate lesions | Severe lesions |
| --- | --- | --- | --- |
| Center 1 | 21 | 11 | 16 |
| Center 2 | 21 | 18 | 14 |
| Center 3 | 14 | 2 | 3 |
| Center 4 | 11 | 14 | 4 |
| Center 5 | 15 | 10 | 4 |
| Center 6 | 2 | 1 | 1 |
| Center 7 | 8 | 5 | 5 |
| Center 8 | 6 | 5 | 7 |
| Center 9 | 0 | 0 | 1 |

Pearson chi2(16) = 21.3414 Pr = 0.166
