# Supplementary material for: Ischemic injury of the upper gastrointestinal tract after out-of-hospital cardiac arrest: a prospective, multicenter study
Source: Crit Care. 2022 Mar 14;26:59. doi: 10.1186/s13054-022-03939-9 (PMC8919548; doi:10.1186/s13054-022-03939-9)
Supplement: Supplementary file 4 — Additional file 4. Changes in SOFA score at specific time points during the ICU stay in the different group. [file 13054_2022_3939_MOESM4_ESM.docx]

**Additionale File 4**

**of the study by Grimaldi et al.**

**Ischemic injury of the upper digestive tract after out-of-hospital cardiac arrest: a prospective, multicentre study**

Changes in SOFA score at specific time points of ICU admission according to the presence and severity of gastrointestinal ischemic lesions

|  | D1 | D2 | Inclusion | D5 | D8 |
| --- | --- | --- | --- | --- | --- |
| No lesion | 0 (ref) | 0 (ref) | 0 (ref) | 0 (ref) | 0 (ref) |
| Moderate lesions | -0.12  [-1.14;0.90] | 0.003  [-1.08;1.09] | 0.13  [-0.95;1.21] | -0.85  [-2.10;0.40] | 0.18  [-1.36;1.72] |
| Severe lesions | 0.52  [-0.56;1.59] | 0.94  [-0.23;2.11] | **1.57 [0.40;2.74]** | 0.96  [-0.43;2.35] | **2.17 [0.43;3.91]** |

Analyses of the change in SOFA score at different time points were based on mixed linear regression models with random intercepts for repeated measurements. Factors associated with the outcome were identified using univariate mixed linear regression models. The results are expressed as regression coefficients (β) with 95% confidence intervals. A positive coefficient indicate a higher sofa score in this category as compared to the reference. CI95% that don’t include 0 indicate significant results (in bold in the table).
