# Supplementary material for: Ischemic injury of the upper gastrointestinal tract after out-of-hospital cardiac arrest: a prospective, multicenter study
Source: Crit Care. 2022 Mar 14;26:59. doi: 10.1186/s13054-022-03939-9 (PMC8919548; doi:10.1186/s13054-022-03939-9)
Supplement: Supplementary file 5 — Additional file 5. Evolution of SOFA score according to severity of gastrointestinal ischemic lesions. [file 13054_2022_3939_MOESM5_ESM.docx]

**Additional File 5 of the study by Grimaldi et al.**

**Ischemic injury of the upper digestive tract after out-of-hospital cardiac arrest: a prospective, multicentre study**

Evolution of SOFA score according to upper gastrointestinal ischemic lesions severity


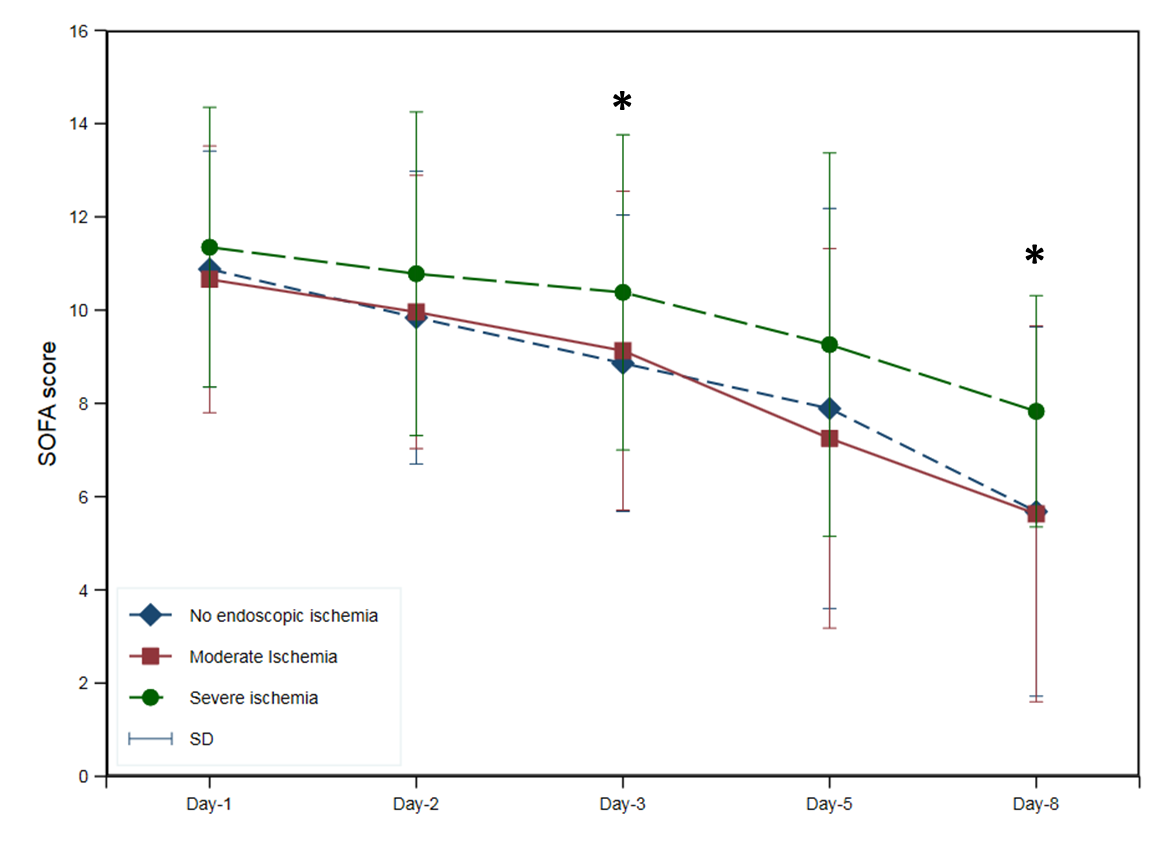


* indicate that SOFA score of patients with severe ischemic lesions is higher than patients without ischemic lesions (reference). Analyses of the change in SOFA score over time were based on mixed linear regression models with random intercepts for repeated measurements
